# Detection of *Streptococcus gallolyticus* and Four Other CRC-Associated Bacteria in Patient Stools Reveals a Potential “Driver” Role for Enterotoxigenic *Bacteroides fragilis*

**DOI:** 10.3389/fcimb.2022.794391

**Published:** 2022-03-11

**Authors:** Bruno Périchon, Julian Lichtl-Häfele, Emma Bergsten, Vincent Delage, Patrick Trieu-Cuot, Philippe Sansonetti, Iradj Sobhani, Shaynoor Dramsi

**Affiliations:** ^1^ Institut Pasteur, Unité de Biologie des Bactéries Pathogènes à Gram-Positif, Paris, France; ^2^ Centre National de la Recherche Scientifique (CNRS) UMR6047, Paris, France; ^3^ Service de Gastroentérologie, Hôpital Henri Mondor, Assistance Publique-Hôpitaux de Paris, Créteil, France; ^4^ Molecular Microbial Pathogenesis Unit, Institut Pasteur; Chaire de Microbiologie et Maladies Infectieuses, Collège de France, Paris, France; ^5^ The Center for Microbes, Development and Health, Institut Pasteur of Shanghai, Chinese Academy of Sciences, Shangaï, China

**Keywords:** *Streptococcus gallolyticus*, pks island, *Bacteroides fragilis bft*+, *Parvimonas micra*, *Fusobacterium nucleatum*, colon cancer, adenomas, adenocarcinomas

## Abstract

**Purpose:**

*Streptococcus gallolyticus* subspecies *gallolyticus* (*SGG*) is an opportunistic pathogen causing invasive infections in the elderly often associated with colon neoplasia. The prevalence of *SGG* in the stools of patients with normal colonoscopy (control) was compared with patients with colorectal adenomas (CRA) or with carcinomas (CRC) from stages I to IV. The presence of the *pk*s island encoding colibactin as well as other CRC-associated bacteria such as toxicogenic *Bacteroides fragilis*, *Fusobacterium nucleatum*, and *Parvimonas micra* was also investigated.

**Patients and Methods:**

Fecal samples collected in France between 2011 and 2016 from patients with normal colonoscopy (*n* = 25), adenoma (*n* = 23), or colorectal cancer at different stages (*n* = 81) were tested by PCR for the presence of *SGG*, *B. fragilis*, *F. nucleatum*, *P. micra*, and the *pks* island. Relative quantification of *SGG*, *F. nucleatum*, and *P. micra* in stools was performed by qPCR.

**Results:**

*SGG* prevalence was significantly increased in the CRC group. Our results also revealed i) a strong and significant increase of toxinogenic *B. fragilis* in patients with early-stage adenoma and of *pks* island at late-stage CRC and ii) increased levels of *F. nucleatum* and *P. micra* in the stools of CRC patients. Furthermore, the simultaneous detection of these five bacterial markers was only found in CRC patients.

**Conclusions:**

Our results indicate that the prevalence or relative levels of CRC-associated bacteria vary during CRC development. Among them, *B. fragilis* (*bft*+) was singled out as the sole pathobiont detected at the early adenoma stage.

## Introduction

The human colon is a complex symbiotic organ where bacteria achieve the highest density, referred to as the gut microbiome. Thousands of bacterial species colonizing the host mucosal surface are commensal symbionts, shaping the host immune system, participating in the host metabolism, and providing a barrier against colonization by potential pathogenic bacteria. Colorectal cancer (CRC) is the second most common cause of cancer mortality worldwide with more than 1.9 million new cases and more than 900,000 deaths per year (Bray et al., 2018; Xi and Xu, 2021). The etiology of CRC is heterogeneous and multifactorial. CRC arises from the accumulation of genetic and epigenetic alterations over a period of 10–15 years leading to a typical adenoma–carcinoma sequence (Fearon and Vogelstein, 1990). Recent progress in high-throughput sequencing revealed a link between the risk of colorectal cancer in humans and modifications of the composition of the gut microbiome referred to as dysbiosis (Sobhani et al., 2011; Nakatsu et al., 2015; Gagnière et al., 2016). In 1974, a seminal study demonstrated that germ-free rats develop much fewer tumors compared with the same rats with normal flora (Reddy et al., 1974). Equally important, mice transplanted with fecal microbiota from either patients with CRC or mice carrying the Apc^Min^ mutation develop more intestinal polyps than mice receiving microbiota from healthy volunteers or from control animals (Wong et al., 2017). It is now widely accepted that colonic microbiota dysbiosis participates in the oncogenic process with a higher relative abundance of potentially pro-carcinogenic bacteria, including *Fusobacterium nucleatum*, *Parvimonas micra*, *Escherichia coli pks*+, toxinogenic *Bacteriodes fragilis*, and *Streptococcus gallolyticus* (Janney et al., 2020).

The first report linking *S. gallolyticus* subsp. *gallolyticus* (*SGG*) to CRC was published in 1951 (McCOY and Mason, 1951). In this early study, *SGG* was referred to as *Streptococcus bovis* biotype I. A reclassification of this large group of bacteria belonging to group D streptococci has been proposed based on molecular tools (Poyart et al., 2002; Schlegel et al., 2003). *Streptococcus gallolyticu*s is now subdivided into three subspecies, subsp. *gallolyticus*, subsp. *pasteurianus*, and subsp. *macedonicu*s. Only *SGG* is associated with CRC. Intestinal carriage of *S. gallolyticus* in humans has been estimated between 2.5% and 15% by culture techniques (Klein et al., 1977; Dubrow et al., 1991; Chirouze et al., 2013; Paritsky et al., 2015). However, a study enrolling 99 healthy volunteers in Germany using real-time PCR indicates a much higher detection rate (62.5%) (Dumke et al., 2017).

In this work, we aimed at determining the prevalence of *SGG* in the stools of French patients with adenomas or carcinomas at various stages as compared with a control group of patients with normal colonoscopies. In addition, we also tested the presence of other “suspects” such as *B. fragilis bft+* (ETBF), *E. coli* pks+, *F. nucleatum*, and *P. micra*.

## Patients and Methods

### Recruitment of Participants and Collection of Samples

Patients referred to university hospitals for colonoscopy were enrolled in several cohorts. All individuals underwent colonoscopy due to symptoms or due to a positive fecal blood test (FOBT). All cohort studies were registered on ClinicalTrials.gov (NCT01270360). The study protocol was approved by the Ethics Committee of Comité de Protection des Personnes Paris Est-Henri Mondor (no. 10-006 in 2010). All participants signed an informed consent. The exclusion criteria for these cohorts were a history of colorectal surgery due to CRC, familial adenomatous polyposis, Lynch syndrome, infection, inflammatory bowel disease, and exposure to antibiotics during the 3 weeks preceding the colonoscopy. For more details, see (Sobhani et al., 2019) and **Table S1**.

### Patient Population

We first used a collection of 74 fecal samples from patients with normal colonoscopy (*n* = 25), colorectal adenoma (CRA, *n* = 23), and colorectal cancer (CRC, *n* = 26), collected at the Henri Mondor Hospital between 2011 and 2016 (VATNIMAD collection). CRA patient colonoscopies were indicative of early stages with benign tumors including small hyperproliferations and small and intermediate adenomas, but no severe dysplasia (precancerous polyps). Twenty-three CRC patients at stages I and II, according to the Union of International Cancer Control (UICC) classification (O’Sullivan et al., 2017), displayed various stages of the primary tumor but no signs of lymph node invasion or metastasis. We completed the initial collection by adding fecal samples from 37 patients with stage III CRC (DETECT, CCR, and ECKINOXE collections) and 18 patients with stage IV CRC (DETECT and CLIMAT collections). Finally, another 85 additional fecal samples of patients with normal colonoscopies (VATNIMAD and CCR collections) were included to measure the prevalence of *S. gallolyticus* subsp. *gallolyticus* in a larger control population.

### Fecal Samples and Bacterial DNA Extraction

Whole fresh stools were collected in sterile boxes, and within 4 h, 10 g were frozen at –20°C, for analysis. All the fecal samples included here were chosen at baseline prior to therapy. Bacterial DNA was extracted from aliquots of feces. DNA isolation was performed using the Promega Wizard^®^ Genomic DNA Purification Kit, following the modified protocol from Ahlroos and Tynkkynen in 2009 (Ahlroos and Tynkkynen, 2009). Total DNA was extracted from 150 mg of stools. Quantification of extracted DNA was measured with the “Qubit 2 Fluorometer”.

### Detection of Bacteria by PCR

PCRs were conducted in a 2720 Thermal Cycler (Applied Biosystems, Foster City, USA) using primers specific for *S. gallolyticus* (*sodA1/sodA2*) (Sasaki et al., 2004), *P. micra* (16S rDNA) (Eick et al., 2011), *B. fragilis* (16S rDNA) (Tong et al., 2011), *F. nucleatum* (16S rDNA) (Boutaga et al., 2005), *clbN* implicated in colibactin polyketide synthesis (Johnson et al., 2008), and *bft* encoding a toxin of *B. fragilis* (**Table S2**). Amplifications were carried out with the following thermal cycling profiles: 3 min at 95°C; 40 cycles of amplification consisting of 10 s at 94°C, 15 s at 55°C, and 25 s at 72°C; and 3 min at 72°C for the final extension. DNA fragments of correct sizes were identified on agarose gels.

### Relative Quantification of Bacteria Species by qPCR

Quantitative real-time PCR was conducted in a CFX96 Touch™ Real-Time PCR Detection System (Bio-Rad) using the same oligonucleotide primers except for *S. gallolyticus* (*sodA5/sodA6*) (**Table S2**). DNA fragments of the expected size were identified by melt temperature peak analysis. Relative quantification was calculated using the following ΔCt formula: Rq = log_10_(2^−(CtBacteria − CtAllBact)^).

### Identification of the *Streptococcus gallolyticus* Subspecies

The sensitivity of *sodA* primers was first evaluated by artificial contamination of a fecal sample with various amounts of *SGG* (from 1.25 * 10^9^ to 1.25 * 10^2^ CFU/100 mg feces). After DNA extraction of the stools, we found that *sodA1/sodA2* primers allowed the detection of 1.25 * 10^2^ CFU/100 mg feces (data not shown) and were used hereafter for *SGG* detection in our human stool collection.

Differences in the *sodA* DNA sequence were used to distinguish the three *S. gallolyticus* subspecies, which were fully sequenced in all *S. gallolyticus-*positive samples.

### Statistical Analysis

The *χ* ^2^ test or the Fisher’s exact test was used to compare bacterial proportions between each group of patients. The non-parametric Mann–Whitney *U* test was used to compare differences in continuous variables between groups. Results were considered statistically significant when *p*-values were <0.05.

## Results

### Patient Characteristics

The characteristics of individuals included in this study are summarized in **Table 1** and details can be found in **Table S1**. The cohort was composed of 80 men (62%) and 49 women (38%). The average age was 63.5 ± 7.9 years and the mean body mass index (BMI) is 25.2 ± 5.8. The study included 25 individuals with normal colonoscopy (13 men, 12 women, ratio 1.1:1.0), 23 patients with benign adenomas (the CRA group consisting of 14 men and 9 women, ratio 1.6:1.0), 26 patients with colorectal cancer at early stages I/II (17 men, 9 women, ratio 1.9:1.0), 37 patients with CRC at stage III (24 men, 13 women, ratio 1.8:1.0), and 18 patients with CRC at stage IV (12 men, 6 women, ratio 2.0:1.0). We noted a higher incidence of CRC in men compared with the general population. The gender ratio (1.9:1.0) in the global CRC group (including stages I to IV) reflected the higher CRC incidence observed among men in Western Europe (Rawla et al., 2019).

**Table 1:** Characteristics of patients included in this study.

	Total	Control	CRA	CRC			
				Global	Stage I/II	Stage III	Stage IV
Number of samples	129	25	23	81	26	37	18
Men (*n*)	80	13	14	53	17	24	12
Women (*n*)	49	12	9	28	9	13	6
Mean age (years)	63.5	63.7	61.4	64.1	67.4	63.2	61.2
Mean age: men (years)	63.1	62.6	60.5	63.9	66.3	64.8	59.0
Mean age: women (years)	64.2	64.3	62.8	64.3	69.2	60.3	65.7
Mean BMI^a^	25.2	26.0	26.1	24.6	26.9	21.8	27.2
Mean BMI: men	24.8	24.9	26.1	24.4	25.7	22.3	26.5
Mean BMI: women	25.8	27.0	26.2	25.1	29.1	20.9	28.7

^a^BMI, body mass index.

### Detection of *Streptococcus gallolyticus* subsp. *gallolyticus* in Human Stools

Detection of *S. gallolyticus* was performed by PCR using oligonucleotide primers specific for the *sodA* gene followed by DNA sequencing for subspecies identification, i.e., *gallolyticus*, *pasteurianus*, or *macedonicus* as previously described (Sasaki et al., 2004).

*SGG* was detected at about 30% in normal (*n* = 25) and CRA (*n* = 23) stools and at about 50% in CRC stools from stages I/II to IV (*n* = 81) (**Table 2**). Since our initial control cohort sample was low (*n* = 25), we tested the prevalence of *SGG* in two other cohorts composed of 50 (30 women, 20 men) and 35 (24 women, 11 men) individuals with normal colonoscopy. We found 28 positive samples over a total of 85 samples (32.9%). This result is in perfect agreement with our initial result (8/25, 32%) (**Table 2**). Taken together, the prevalence of *SGG* in our control population (36 positive over 110) is 32.5%.

**Table 2:** Detection of *Streptococcus gallolyticus* subsp. *gallolyticus* (SGG), *Fusobacterium nucleatum* (FN), *Bacteroides fragilis*, enterotoxigenic *B. fragilis* (ETBF), *Parvimonas micra* (PM), and *pks* island in the stools from CRC, CRA, and control patients using the PCR assay.

Positive detection of:	Total (*N* = 129)		Control (*N* = 25)		CRA (*N* = 23)		CRC							
							Global		CRC I+II		CRC III		CRC IV	
							(*N* = 81)		(*N* = 26)		(*N* = 37)		(*N* = 18)	
	*n*	%	*n*	%	*n*	%	*n*	%	*n*	%	*n*	%	*n*	%
*SGG*	56	43.4	8	32.0	7	30.4	41	50.6	13	50.0	19	51.4	9	50.0
FN	82	63.6	12	48.0	13	56.5	57	70.4	16	61.5	28	75.7	13	72.2
BF	81	62.8	12	48.0	19	82.6	50	61.7	16	61.5	25	67.6	9	50.0
ETBF	44	34.1	6	24.0	13	56.5	25	30.9	9	34.6	12	32.4	4	22.2
PM	103	79.8	18	72.0	15	65.2	70	86.4	22	84.6	32	86.5	16	88.9
*pks* island	53	41.1	6	24.0	7	30.4	40	49.4	11	42.3	16	43.2	13	72.2

The results in **Table 2** together with those obtained above show that the detection rate of *SGG* is not different in CRA stools (7/23; 30.4%) but is increased significantly (*p* = 0.02) in CRC samples at all stages (41/81, 50.6%) as compared with the global control group (36/110, 32.5%).

### Prevalence of *Bacteroides fragilis*, *Fusobacterium nucleatum*, and *Parvimonas micra*

Detection of *B. fragilis*, *F. nucleatum*, and *P. micra* was carried out on the same samples by PCR and shown in **Table 2**.

**Figure 1:**
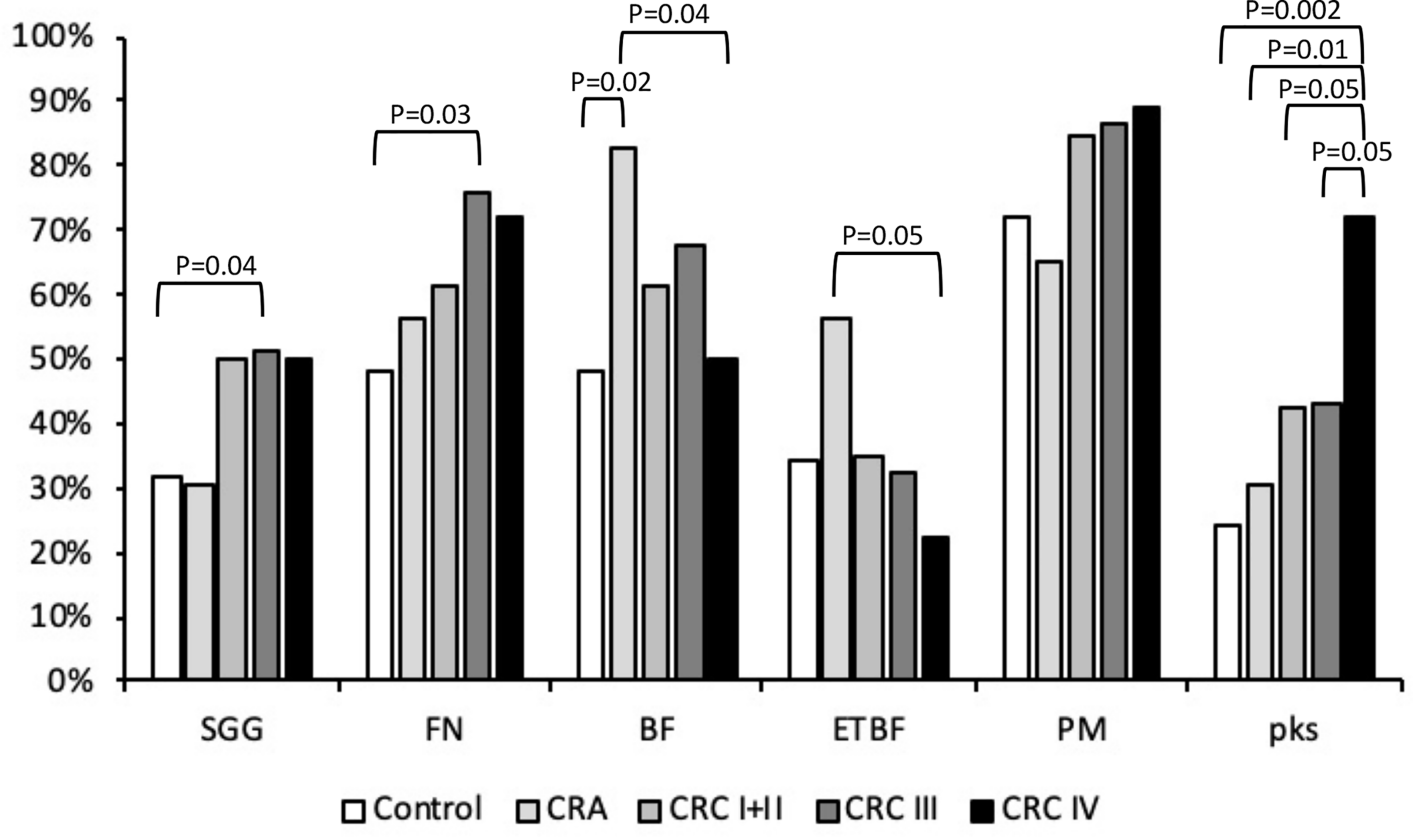
Presence of *Streptococcus gallolyticus* (*SGG*), *Fusobacterium nucleatum* (FN), enterotoxigenic *Bacteroides fragilis* (ETBF), and *Parvimonas micra* (PM) *pks* gene in feces. The presence of bacterial DNA was tested by PCR using specific primers (**Table 1**). Statistical significance was calculated using the *χ* ^2^ test or Fisher’s exact test (when *n* < 5). The real *p*-value is indicated for groups displaying significant differences.

*Bacteroides fragilis* is a member of Bacteroidetes, a major phylum of the human gut microbiota. *Bacteroides fragilis* is considered as a commensal bacterium, whereas enterotoxigenic *B. fragilis* (ETBF) synthesizing fragilysin (*bft* ^+^) has been strongly associated with CRC. *Bacteroides fragilis* was detected in 62.8% of our stool collection (**Table 2**). It was detected in 19/23 CRA samples (83%) but only in approximately 62% of the CRC samples and 48% of the control stools (**Table 2**), indicating a solid association between *B. fragilis* and CRA (CRA vs. control, *p* = 0.02). To determine the type (non-toxinogenic or toxinogenic) of *B. fragilis* present in our samples, detection of the *bft* gene encoding *B. fragilis* toxin was carried out by PCR using specific primers (**Table S2**). As shown in **Table 2**, the presence of ETBF was significantly increased in the stools from CRA patients (56.5%) as compared with the control (24%, *p* < 0.05) and CRC groups (31%, *p* = 0.06) (**Table 2**, **Figure 1**). Interestingly, ETBF detection decreased during the evolution of CRC, from 34.6% in CRC stage I/II to 22.2% in CRC stage IV (**Table 2**, **Figure 1**), with a significant statistical difference (*p* < 0.05) between CRA and CRC stage IV.

*Fusobacterium* is a Gram-negative aerobic bacillus commensal of the human mouth and gastrointestinal and urogenital tracts. *Fusobacterium nucleatum* was detected by PCR and its presence steadily increased: 48% in the control group, 56.5% in the CRA samples, and 70.4% in the CRC group (**Table 2** and **Figure 1**) (CRC III vs. controls, *p* < 0.05). *Fusobacterium nucleatum* detection increased, although this was not statistically significant, at later stages of CRC (III/IV) as compared with earlier stages I/II (74.5% vs. 61.5%, *p* = 0.2, NS).

*Parvimonas micra* is a Gram-positive anaerobic coccus of the oral flora associated with CRC (Wu et al., 2013; Yu et al., 2017). As shown in **Table 2** and **Figure 1**, we could not detect any significant difference in the carriage of *P. micra* in the stools of our control, CRA, and CRC patients. However, a slight increase of the bacterial load in the CRC group (86.4%) was observed as compared with the control (72%) or CRA (65.2%) groups (*p* = 0.09, NS).

### Prevalence of the *pks* Island

Colibactin is a genotoxin causing DNA double-strand breaks in eukaryotic cells. It is synthesized by a non-ribosomal polyketide synthase (pks) assembly line consisting of 19 genes (*clbA* to *clbS*) located on a 54-kb genomic island. Detection of the *pks* island was performed by PCR of the *clbN* gene often used as a marker for the 3′ region of this island (Johnson et al., 2008). Overall, *clbN* was detected with a higher prevalence in CRC stools (49.4%) compared with the non-symptomatic (control 24%, *p* < 0.005) or CRA groups (30.4%, *p* < 0.01) (**Table 2** and **Figure 1**). Analysis of *pks* prevalence in our global cohort revealed a progressive increase during CRC evolution peaking at the latest stage (72.2% CRC stage IV) as compared with earlier stages, i.e., CRC stage I/II (42.3%, *p* < 0.05) and CRC stage III (43.2%, *p* < 0.05) (**Table 2** and **Figure 1**).

To test the association between the presence of a specific bacterial species and CRC, we used the *χ* ^2^ test for trends with the presence/absence of bacteria as the binary variable and controls, adenoma, CRC I/II, CRC III, and CRC IV as the ordered categorical variables. Results indicate a statistical significance for *F. nucleatum* (*p* < 0.05) and the *pks* island (*p* < 0.005).

### Relative Quantification of *Streptococcus gallolyticus*, *Fusobacterium nucleatum*, and *Parvimonas micra* in Stools

**Figure 2:**
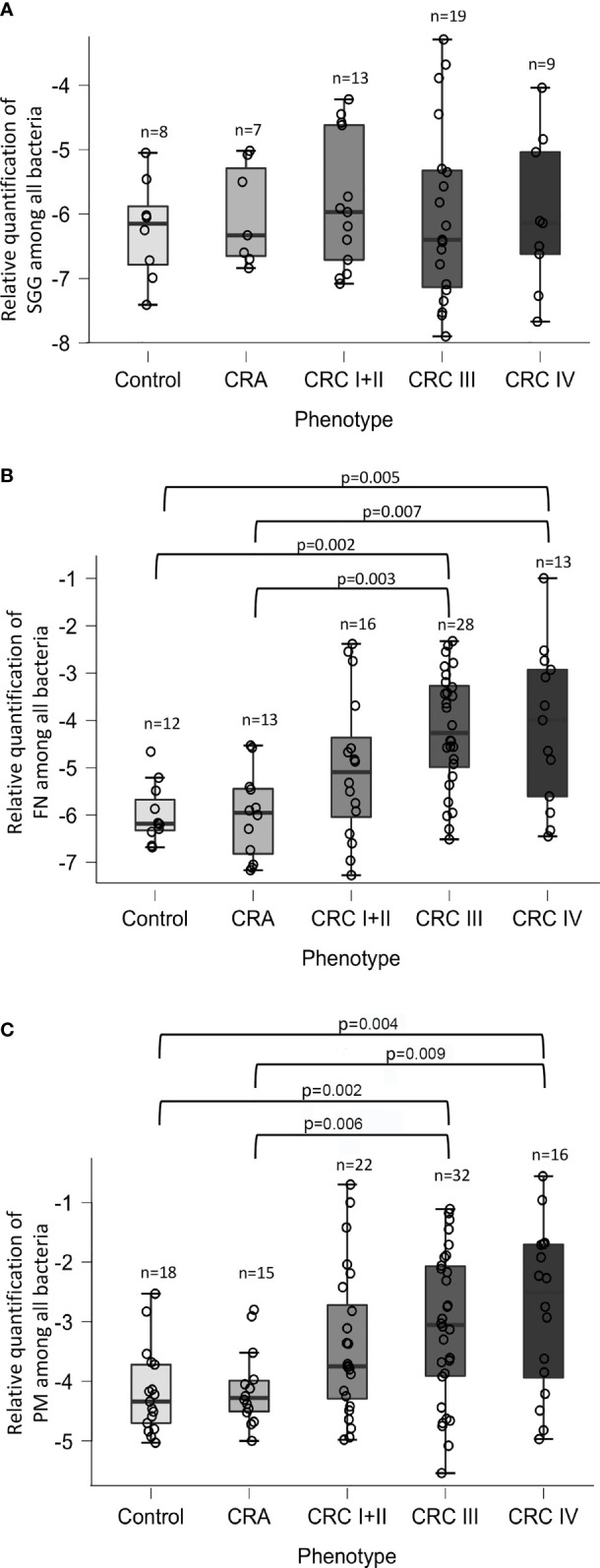
Relative quantification of *Streptococcus gallolyticus* (*SGG*), *Fusobacterium nucleatum* (FN), and *Parvimonas micra* (PM). The levels of **(A)** *SGG*, **(B)** FN, and **(C)** PM in control, adenomatous (CRA), or patients at each stage of colorectal cancer (stage I or II, stage III or IV) were calculated as a relative quantification of bacteria among total bacteria [log_10_(2^−(CqBacteria − CqAllBacteria)^)] using the 16S rRNA gene as a reference. Statistical significance was calculated using the non-parametric Mann–Whitney *U* test. The real *p*-value is indicated for groups displaying significant differences.

We next decided to determine the relative quantity of three bacteria in our CRC cohort. Real-time qPCR assays were performed by using primers specific for *S. gallolyticus*, *F. nucleatum*, or *P. micra* (**Table S2**) and universal 16S rRNA primers (Allbact, **Table S2**) to determine the total number of bacteria present in the stool sample. Relative quantification of *SGG* revealed no differences in abundance in control, CRA, and CRC cohort, nor during CRC development (**Figure 2A**). In contrast, the respective abundance of *F. nucleatum* (**Figure 2B**) and *P. micra* (**Figure 2C**) was statistically increased in CRC patients as compared with CRA or controls. Detailed results indicated that the relative abundance of *F. nucleatum* and *P. micra* increased during the development of cancer, notably at later stages (**Figures 2B, C** **)**.

### Presence of Several Bacterial Markers in CRC Stools

**Figure 3:**
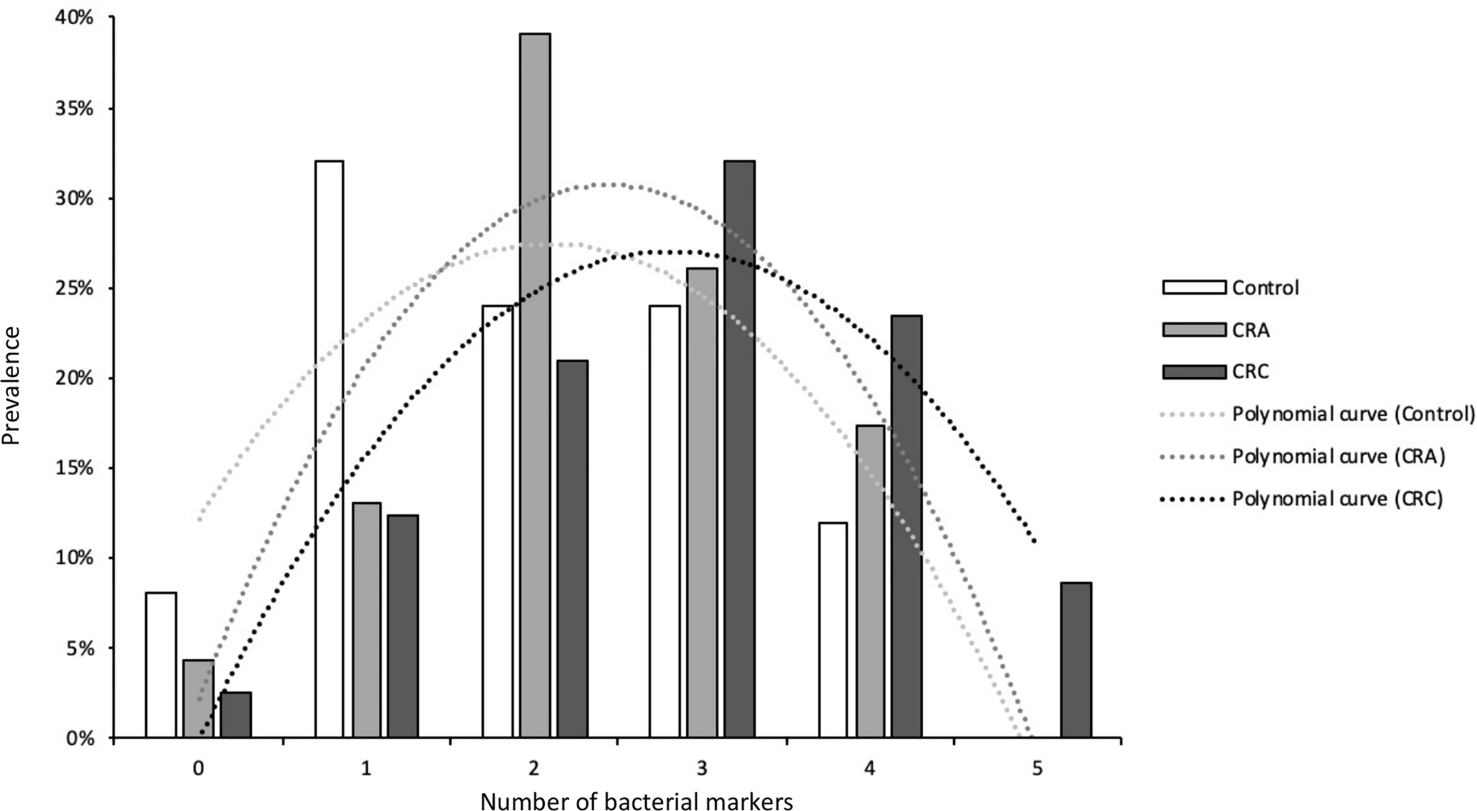
The number of suspects bacterial markers found in the three groups: control, CRA, and CRC patients.

As shown in **Figure 3**, a clear shift is observed in the number of suspicious bacterial markers detected in patients, increasing progressively with the evolution of the disease. This result clearly indicates that multiple opportunistic bacteria contribute to the progression of CRC. Hence, the development of potential fecal diagnostic tools for CRC detection should be based on at least five bacterial markers [*SGG*, *F. nucleatum* (FN), *Parvimonas micra* (PM), ETBF, and *pks*).

## Discussion

Genetic and epigenetic modifications are of major concern in CRC development. It is now well established that dysbiosis of the gut microbiota contributes to the progression of colonic carcinogenesis. The development of new detection methods (16S rRNA gene sequencing, metagenomics, transcriptomics, proteomics, metabolomics) has led to an incredible improvement in defining the composition and function of the intestinal microbiome. However, microbiome variations along the colonic axis and between intraluminal and mucosal microbe communities have made the creation of a complete species catalog difficult (Sobhani et al., 2011). Nevertheless, several bacteria such as colibactin-producing *E. coli*, ETBF, *SGG*, and two oral bacteria, *F. nucleatum* and *P. micra*, appear to be strongly associated with colorectal cancer (Reddy et al., 1974; Fearon and Vogelstein, 1990). The molecular mechanisms underlying the promotion of colonic cell proliferation have not been completely elucidated until now. A bacterial “driver–passenger model” has been proposed in which CRC would be initiated by “driver” bacteria that cause changes in the tumor microenvironment allowing the colonization by “passenger” bacteria that could promote CRC progression (Tjalsma et al., 2012).

We found a prevalence of 32.5% for *SGG* in a French cohort of 110 patients with normal colonoscopies, which contrasts with the prevalence of 62.5% in the stools of 99 healthy volunteers in Germany (Dumke et al., 2017). Age and geographical distribution affecting microbiota composition could be the main factors explaining the difference between these two studies. Indeed, the mean age was 48.4 ± 14.9 in Dumke’s study, whereas it was 63.5 ± 7.9 years in our study. Our results demonstrating a higher prevalence but not a higher charge of *SGG* in CRC independent of the stage of CRC are more in favor of a “passenger” role for *S. gallolyticus* subsp. *gallolyticus* in CRC. This result is in line with our previous study indicating that *SGG* is a passenger bacterium taking advantage of tumoral conditions to outcompete commensals of the gut microbiota such as *Enterococcus faecalis* (Aymeric et al., 2018).

In agreement with metagenomics data showing a strong enrichment of *F. nucleatum* (FN) in the microbiota of CRC patients, we also observed a significant increase of FN in the stools of patients with CRC, especially at later stages. The bacterial adhesin Fap2 has been shown to bind specifically to colon tumoral tissues expressing high levels of Gal-Gal-NAc (Abed et al., 2016), while a recent study points to a role of FN in the adenoma–carcinoma transition (Bundgaard-Nielsen et al., 2019). Of note, a study on the Iranian population reports high numbers of *F. nucleatum*, *S. bovis*/*gallolyticus*, ETBF, and *F. nucleatum* in tubular adenomas and polyps, especially in villous/tubulovillous polyps, as compared with the samples from normal, hyperplastic, and sessile serrated polyps by quantitative real-time PCR (Rezasoltani et al., 2018).

The role of *P. micra* in CRC remains unknown, but it has been shown that it can promote intestinal carcinogenesis in APC^min/+^ mice (Yu et al., 2019). Here, we demonstrated that the relative abundance of *P. micra* and *F. nucleatum* is statistically higher in CRC stools, as previously shown (Liang et al., 2017; Dai et al., 2018; Xu et al., 2020; Löwenmark et al., 2020). These results indicate that bacterial quantification is an important element for stool-based CRC diagnostics.

Enterotoxigenic *B. fragilis* is a gut pathobiont producing a potent toxin (BFT) that cleaves the adherens junction protein E-cadherin, altering the intestinal barrier and causing diarrhea. Alteration of E-cadherin/beta-catenin interactions activates the Wnt pathway leading to cell proliferation. Indeed, ETBF has been proposed to be a driver bacterium (Tjalsma et al., 2012). Our results strongly support this hypothesis since the prevalence of BFT was strongly increased in CRA stool samples as compared with the control or CRC groups (**Figure 1**). Interestingly, *bft* presence decreased progressively during later stages of CRC indicating a transient role for this bacterium during transition from adenoma to carcinoma. However, these results need to be confirmed in a larger cohort especially with more samples from patients with colorectal adenomas.

Colibactin is another bacterial toxin strongly associated with CRC. The structure of colibactin and its DNA cross-links has been solved recently (Xue et al., 2019), and importantly, colibactin DNA-damage signatures have been found in CRC patients (Dziubańska-Kusibab et al., 2020). Colibactin is produced by the *pks* island found essentially in group B2 *E. coli*, but also in other *Enterobacteriaceae* such as *Klebsiella pneumoniae*, *Enterobacter aerogenes*, *Citrobacter koseri*, and group B1 *E. coli* isolates. We found that the prevalence of the *clbN* gene in DNA extracted from patient stools was mainly high in CRC samples, especially at stage IV. These results suggest a role for colibactin at terminal stages in the development of CRC. It is worth mentioning that bacterial biofilms composed mainly of *E. coli* pks+ and ETBF are found in the mucosa of patients with familial adenomatous polyposis (FAP) and that a synergistic effect of the two toxins (*bft* and *pks*) driving the development of colon tumor was demonstrated in ApcMin^D716/+^ (Dejea et al., 2018). Additional results from this study also indicate that *Bacteroides* precedes and promotes the adherence of pks+ *E. coli*, which agrees with our data.

Altogether our results support the notion that colorectal cancer development is not caused by a single pathogen but is probably driven by several pathobionts acting together or sequentially during the evolution of CRC. In line with this idea, detection and quantification of five suspect markers in fecal samples may be necessary to develop a robust non-invasive diagnostic tool (Yu et al., 2017; Liang et al., 2017).

## Data Availability Statement

The raw data supporting the conclusions of this article will be made available by the authors, without undue reservation.

## Ethics Statement

The study protocol was approved by the Ethics Committee of Comité de Protection des Personnes Paris Est-Henri Mondor (no. 10-006 in 2010). All participants signed an informed consent. The patients/participants provided their written informed consent to participate in this study.

## Author Contributions

BP, JL-H, and VD performed the experimental work. EB and IS provided the patients’ stools. IS and PS supervised the writing of the manuscript. PT-C helped in funding acquisition. JL-H and SD designed the study. BP and SD analyzed the data and organized and wrote the manuscript. All authors contributed to the article and approved the submitted version.

## Funding

This work was supported by the Institut National contre le Cancer (INCA, grant PLBIO16-025) and the French Government’s Investissement d’Avenir program, Laboratoire d’Excellence Integrative Biology of Emerging Infectious Diseases (grant no. ANR-10-LABX-62- IBEID). We also thank SNFGE, the French Society of Gastroenterology, for their financial support.

## Conflict of Interest

The authors declare that the research was conducted in the absence of any commercial or financial relationships that could be construed as a potential conflict of interest

## Publisher’s Note

All claims expressed in this article are solely those of the authors and do not necessarily represent those of their affiliated organizations, or those of the publisher, the editors and the reviewers. Any product that may be evaluated in this article, or claim that may be made by its manufacturer, is not guaranteed or endorsed by the publisher.
