# Supplementary material for: Detection of Streptococcus gallolyticus and Four Other CRC-Associated Bacteria in Patient Stools Reveals a Potential “Driver” Role for Enterotoxigenic Bacteroides fragilis
Source: Front Cell Infect Microbiol. 2022 Mar 11;12:794391. doi: 10.3389/fcimb.2022.794391 (PMC8963412; doi:10.3389/fcimb.2022.794391)
Supplement: Supplementary file 1 [file Table_1.docx]

**Table S1.** Patient characteristics. localization of tumors. and presence of five bacterial markers in stools of control subjects. CRA and CRC (stage I/II. III. and IV) patients.

| **Ref** | **Phenotype** | **Sex** | **BMI** | **Age** | **Localization** | **SGG** | **FN** | **ETBF** | **PM** | ***pks*** |
| --- | --- | --- | --- | --- | --- | --- | --- | --- | --- | --- |
| 1 | Control | F | 23.5 | 60 | NA |  |  |  | **+** |  |
| 2 | Control | F | 42.8 | 62 | NA |  | **+** |  | **+** |  |
| 3 | Control | F | 18.3 | 74 | NA |  | **+** |  | **+** | **+** |
| 4 | Control | F | 30.0 | 59 | NA | **+** | **+** | **+** | **+** |  |
| 5 | Control | F | 33.3 | 67 | NA | **+** | **+** | **+** |  |  |
| 6 | Control | F | 38.6 | 65 | NA | **+** |  |  | **+** |  |
| 7 | Control | M | unk | 58 | NA |  |  |  |  |  |
| 8 | Control | F | 27.5 | 61 | NA | **+** | **+** | **+** |  |  |
| 9 | Control | F | 21.6 | 71 | NA |  | **+** |  | **+** | **+** |
| 10 | Control | M | 31.8 | 63 | NA | **+** | **+** | **+** | **+** |  |
| 11 | Control | M | 22.6 | 63 | NA |  | **+** |  |  |  |
| 12 | Control | M | 24.5 | 64 | NA |  | **+** |  |  | **+** |
| 13 | Control | M | 25.4 | 51 | NA |  |  |  |  |  |
| 14 | Control | M | 23.7 | 65 | NA |  |  |  |  | **+** |
| 15 | Control | F | 26.1 | 68 | NA |  |  |  | **+** |  |
| 16 | Control | M | 26.3 | 57 | NA |  |  |  | **+** |  |
| 17 | Control | M | 21.7 | 66 | NA |  |  |  | **+** |  |
| 18 | Control | M | 29.0 | 62 | NA |  | **+** |  | **+** |  |
| 19 | Control | F | 21.9 | 64 | NA | **+** |  | **+** | **+** |  |
| 20 | Control | F | 18.0 | 72 | NA | **+** | **+** | **+** | **+** |  |
| 21 | Control | M | 19.4 | 66 | NA | **+** |  |  | **+** |  |
| 22 | Control | M | 26.9 | 62 | NA |  |  |  | **+** | **+** |
| 23 | Control | M | 22.3 | 72 | NA |  |  |  | **+** |  |
| 24 | Control | M | 25.5 | 65 | NA |  |  |  | **+** |  |
| 25 | Control | F | 22.8 | 55 | NA |  | **+** |  | **+** | **+** |
| 26 | CRA | F | 22.9 | 61 | **LC** |  | **+** |  | **+** | **+** |
| 27 | CRA | F | 30.5 | 59 | **LC** |  |  |  | **+** |  |
| 28 | CRA | M | 22.2 | 55 | **Rectum & LC** | **+** |  | **+** | **+** |  |
| 29 | CRA | M | 22.9 | 59 | **LC & TC** |  | **+** |  | **+** |  |
| 30 | CRA | F | 23.4 | 72 | **TC** | **+** | **+** |  |  |  |
| 31 | CRA | M | 24.5 | 59 | **RC** |  | **+** | **+** | **+** | **+** |
| 32 | CRA | M | 23.0 | 69 | **LC** |  |  | **+** | **+** |  |
| 33 | CRA | M | 25.6 | 57 | **LC & RC** |  |  |  |  |  |
| 34 | CRA | F | 29.1 | 61 | **LC** | **+** | **+** | **+** | **+** |  |
| 35 | CRA | M | 24.7 | 62 | **LC** |  |  |  |  | **+** |
| 36 | CRA | F | 25.2 | 62 | **LC** |  | **+** |  | **+** | **+** |
| 37 | CRA | F | 23.3 | 69 | **LC** |  | **+** | **+** |  |  |
| 38 | CRA | M | 26.2 | 69 | **LC & TC** | **+** | **+** | **+** | **+** |  |
| 39 | CRA | M | 31.0 | 57 | **LC** | **+** |  | **+** |  |  |
| 40 | CRA | M | 34.0 | 59 | **LC & TC** | **+** | **+** | **+** |  | **+** |
| 41 | CRA | F | 26.3 | 59 | **RC** | **+** |  | **+** |  |  |
| 42 | CRA | F | 35.5 | 70 | **LC & TC** |  | **+** |  | **+** |  |
| 43 | CRA | M | 27.1 | 59 | **LC** |  |  | **+** | **+** | **+** |
| 44 | CRA | M | 29.3 | 53 | **LC** |  | **+** |  | **+** | **+** |
| 45 | CRA | M | 23.4 | 51 | **LC & TC** |  |  | **+** |  |  |
| 46 | CRA | F | 19.5 | 52 | **LC** |  | **+** |  | **+** |  |
| 47 | CRA | M | 21.7 | 66 | **LC & TC & RC** |  | **+** | **+** | **+** |  |
| 48 | CRA | M | 29.4 | 72 | **LC** |  |  | **+** | **+** |  |
| 49 | CRC I/II | M | 26.3 | 58 | **LC** |  | **+** |  | **+** | **+** |
| 50 | CRC I/II | M | 37.9 | 69 | **LC** | **+** | **+** | **+** | **+** | **+** |
| 51 | CRC I/II | M | 27.8 | 74 | **LC** | **+** | **+** | **+** | **+** |  |
| 52 | CRC I/II | F | 39.1 | 63 | **RC** |  | **+** | **+** | **+** |  |
| 53 | CRC I/II | F | unk | 65 | **LC** | **+** | **+** | **+** | **+** | **+** |
| 54 | CRC I/II | M | 30.0 | 62 | **LC** |  |  | **+** | **+** | **+** |
| 55 | CRC I/II | M | 25.0 | 81 | **RC** |  | **+** |  | **+** |  |
| 56 | CRC I/II | M | 21.6 | 73 | **LC** | **+** | **+** | **+** | **+** |  |
| 57 | CRC I/II | M | 13.5 | 56 | **LC** | **+** | **+** | **+** | **+** |  |
| 58 | CRC I/II | F | 40.6 | 74 | **RC** |  |  |  |  |  |
| 59 | CRC I/II | M | 25.3 | 60 | **RC** |  |  |  | **+** |  |
| 60 | CRC I/II | M | 23.9 | 65 | **LC** | **+** | **+** |  | **+** |  |
| 61 | CRC I/II | F | 20.5 | 75 | **LC** |  | **+** |  | **+** |  |
| 62 | CRC I/II | M | unk | 54 | **RC** |  |  |  |  | **+** |
| 63 | CRC I/II | F | 21.2 | 69 | **RC** |  |  |  | **+** | **+** |
| 64 | CRC I/II | M | 26.4 | 61 | **LC** | **+** |  | **+** | **+** |  |
| 65 | CRC I/II | M | 24.8 | 69 | **LC** |  |  |  |  | **+** |
| 66 | CRC I/II | F | 21.8 | 72 | **LC** |  | **+** |  | **+** | **+** |
| 67 | CRC I/II | M | 20.8 | 60 | **LC** | **+** | **+** |  | **+** | **+** |
| 68 | CRC I/II | M | 28.1 | Unk | **LC** | **+** |  |  | **+** |  |
| 69 | CRC I/II | M | 36.0 | 72 | **LC** | **+** | **+** |  | **+** |  |
| 70 | CRC I/II | F | 31.0 | 63 | **LC** | **+** | **+** | **+** | **+** | **+** |
| 71 | CRC I/II | M | 26.0 | 73 | **RC** |  |  |  |  |  |
| 72 | CRC I/II | F | 31.1 | 75 | **LC** | **+** | **+** |  | **+** |  |
| 73 | CRC I/II | M | 18.6 | 74 | **RC** | **+** |  |  | **+** |  |
| 74 | CRC I/II | F | 27.4 | 67 | **RC** |  | **+** |  | **+** | **+** |
| 75 | CRC III | M | 29.7 | 71 | **RC** |  |  |  | **+** | **+** |
| 76 | CRC III | M | 23.7 | 63 | **LC** |  |  |  | **+** |  |
| 77 | CRC III | M | 23.1 | 51 | **LC** |  |  |  | **+** |  |
| 78 | CRC III | M | 26.8 | 72 | **LC** | **+** | **+** |  |  | **+** |
| 79 | CRC III | F | 32.3 | 62 | **LC** |  | **+** |  | **+** |  |
| 80 | CRC III | M | 22.4 | 56 | **LC** | **+** |  |  | **+** | **+** |
| 81 | CRC III | M | 28.7 | 77 | **RC** |  |  |  | **+** |  |
| 82 | CRC III | F | 26.6 | 57 | **RC** |  | **+** | **+** | **+** | **+** |
| 83 | CRC III | M | 25.1 | 72 | **LC** | **+** | **+** |  | **+** |  |
| 84 | CRC III | F | 27.7 | 68 | **RC** |  | **+** |  |  |  |
| 85 | CRC III | M | 32.8 | 87 | **RC** | **+** | **+** | **+** | **+** |  |
| 86 | CRC III | M | 26.0 | 69 | **RC** | **+** | **+** |  | **+** |  |
| 87 | CRC III | M | 19.5 | 68 | **RC** | **+** | **+** | **+** | **+** |  |
| 88 | CRC III | M | 15.4 | 65 | **RC** | **+** | **+** | **+** | **+** | **+** |
| 89 | CRC III | M | 17.9 | 72 | **LC** | **+** | **+** | **+** | **+** | **+** |
| 90 | CRC III | M | 22.0 | 42 | **LC** | **+** | **+** |  | **+** |  |
| 91 | CRC III | F | 19.0 | 68 | **LC** | **+** | **+** | **+** | **+** |  |
| 92 | CRC III | F | 16.8 | 57 | **TC** | **+** | **+** | **+** | **+** |  |
| 93 | CRC III | M | 16.9 | 57 | **LC** |  | **+** |  | **+** | **+** |
| 94 | CRC III | F | 15.9 | 56 | **LC** |  | **+** |  | **+** |  |
| 95 | CRC III | F | 17.1 | 64 | **LC** |  | **+** |  | **+** | **+** |
| 96 | CRC III | M | 18.6 | 55 | **RC** | **+** | **+** | **+** | **+** |  |
| 97 | CRC III | M | 19.1 | 69 | **TC** | **+** | **+** |  | **+** |  |
| 98 | CRC III | M | 18.2 | 57 | **LC** |  | **+** |  | **+** | **+** |
| 99 | CRC III | F | 19.9 | 53 | **LC** |  | **+** |  | **+** |  |
| 100 | CRC III | F | 19.1 | 41 | **LC** |  |  |  | **+** |  |
| 101 | CRC III | M | 19.0 | 71 | **RC** |  | **+** |  | **+** | **+** |
| 102 | CRC III | F | 20.7 | 67 | **TC** | **+** |  | **+** | **+** | **+** |
| 103 | CRC III | F | 17.1 | 48 | **LC** |  | **+** | **+** | **+** | **+** |
| 104 | CRC III | M | unk | 73 | **LC** | **+** | **+** |  | **+** |  |
| 105 | CRC III | M | 17.3 | 68 | **RC** |  | **+** |  |  |  |
| 106 | CRC III | M | 17.3 | 66 | **LC** | **+** |  |  |  | **+** |
| 107 | CRC III | F | 17.9 | 72 | **LC** | **+** | **+** |  | **+** | **+** |
| 108 | CRC III | F | 21.9 | 71 | **LC** |  | **+** | **+** | **+** |  |
| 109 | CRC III | M | 22.3 | 58 | **RC** | **+** | **+** |  |  |  |
| 110 | CRC III | M | 23.9 | 55 | **LC** |  | **+** |  | **+** | **+** |
| 111 | CRC III | M | 28.1 | 61 | **LC** | **+** |  | **+** | **+** | **+** |
| 112 | CRC IV | M | 33.3 | 42 | **RC** | **+** |  |  |  |  |
| 113 | CRC IV | F | 23.0 | 65 | **RC** | **+** | **+** |  | **+** | **+** |
| 114 | CRC IV | F | 39.7 | 76 | **LC** | **+** | **+** | **+** | **+** | **+** |
| 115 | CRC IV | M | 31.2 | 59 | **LC** |  | **+** | **+** | **+** | **+** |
| 116 | CRC IV | M | 26.6 | 48 | **LC** |  | **+** |  | **+** | **+** |
| 117 | CRC IV | M | 24.2 | 61 | **RC** |  | **+** |  | **+** |  |
| 118 | CRC IV | F | 29.9 | 61 | **LC** |  | **+** |  | **+** | **+** |
| 119 | CRC IV | M | 29.4 | 65 | **LC** | **+** |  |  | **+** | **+** |
| 120 | CRC IV | F | 34.7 | 71 | **RC** |  |  |  | **+** | **+** |
| 121 | CRC IV | F | 20.2 | 52 | **RC** |  | **+** |  | **+** |  |
| 122 | CRC IV | M | 31.2 | 59 | **RC** | **+** |  |  | **+** |  |
| 123 | CRC IV | M | 25.9 | 71 | **LC** | **+** | **+** | **+** | **+** | **+** |
| 124 | CRC IV | M | 16.9 | 70 | **LC** |  | **+** | **+** | **+** | **+** |
| 125 | CRC IV | M | 26.6 | 63 | **LC** | **+** | **+** |  |  |  |
| 126 | CRC IV | M | 19 | 59 | **LC** |  |  |  | **+** | **+** |
| 127 | CRC IV | M | 31.4 | 53 | **LC** | **+** | **+** |  | **+** | **+** |
| 128 | CRC IV | F | 24.9 | 69 | **LC** |  | **+** |  | **+** | **+** |
| 129 | CRC IV | M | 22.4 | 58 | **LC** | **+** | **+** |  | **+** | **+** |

BMI, body mass index

NA, not applicable

LC, Left colon; RC, Right colon; TC, Transverse colon

Unk, unknown

+, Presence
