# Supplementary material for: Detection of Streptococcus gallolyticus and Four Other CRC-Associated Bacteria in Patient Stools Reveals a Potential “Driver” Role for Enterotoxigenic Bacteroides fragilis
Source: Front Cell Infect Microbiol. 2022 Mar 11;12:794391. doi: 10.3389/fcimb.2022.794391 (PMC8963412; doi:10.3389/fcimb.2022.794391)
Supplement: Supplementary file 2 [file Table_2.docx]

**Table S2. Primers used in this study**

| Name | DNA sequence (5' to 3') | Reference |
| --- | --- | --- |
| SodA1 | CAATGACAATTCACCATGA | (21) |
| SodA2 | TTGGTGCTTTTCCTTGTG |  |
| SodA5 | GCTACTTATGTGGCAAATG | This study |
| SodA6 | CCTGAAAAACAAGAACCAAC |  |
| B. fragilis F | TCRGGAAGAAAGCTTGCT | (23) |
| B. fragilis R | CATCCTTTACCGGAATCCT |  |
| F. nucleatum F | GGATTTATTGGGCGTAAAGC | (24) |
| F. nucleatum R | GGCATTCCTACAAATATCTACGAA |  |
| P. micra F | AACGACGATTAATACCGCATGAGACC | (22) |
| P. micra R | CTTCCTCCTATGATACCGTCATTA |  |
| bft F | GAGCCGAAGACGGTGTATGTGATTTGT | (42) |
| bft R | TGCTCAGCGCCCAGTATATGACCTAGT |  |
| clbN F | GTTTTGCTCGCCAGATAGTCATTC | (25) |
| clbN R | CAGTTCGGGTATGTGTGGAAGG |  |
| Allbact F | CGGTGAATACGTTCCCGG | (43) |
| Allbact R | AAGTCGTAACAAGGTAGCCGTA |  |

42. Toprak NU, Yagci A, Gulluoglu BM, Akin ML, Demirkalem P, Celenk T, Soyletir G. A possible role of Bacteroides fragilis enterotoxin in the aetiology of colorectal cancer. *Clin Microbiol Infect* (2006) 12:782–786. doi:10.1111/j.1469-0691.2006.01494.x

43. Suzuki MT, Taylor LT, DeLong EF. Quantitative Analysis of Small-Subunit rRNA Genes in Mixed Microbial Populations via 5′-Nuclease Assays. *Appl Environ Microbiol* (2000) 66:4605–4614. doi:10.1128/AEM.66.11.4605-4614.2000
